# Health system recovery from Covid-19: What policies are health systems using to tackle backlogs and bring down waiting times in the wake of the pandemic?

**DOI:** 10.1093/eurpub/ckac129.212

**Published:** 2022-10-25

**Authors:** S Reed, L Schlepper, N Edwards

**Affiliations:** Nuffield Trust, London, UK

## Abstract

The pandemic has left even the most well-equipped health systems vulnerable and required difficult trade-offs to balance both Covid-19 and non-Covid-19 health services. Across the globe, planned and routine health services have been scaled down during peaks of the crisis to meet the needs of acute and Covid-19-related care, resulting in growing care backlogs and increase in the number of patients waiting for treatment. To identify potential policy solutions, we have consulted the Covid-19 Health System Response Monitor, interviewed experts and analysed recovery strategies in 16 OECD and EU countries. Many country responses display striking similarities despite very real differences in the organisation of health and care services. These include:

1) increasing the supply of workforce by widening the scope of authority for different roles, investing heavily in recruitment and training for key roles, and improving the terms and conditions of work;

2) boosting productivity by introducing financial incentives and targets, reconfiguring facilities to better separate planned and emergency work”, optimising referrals and waiting list management, and outsourcing more care to the private sector; and

3) investing in out-of-hospital alternatives to care, including expanding primary and community care models and developing digital, home care and rehabilitative capacity

Policymakers will need to balance the immediate pressures of clearing backlogs with long-term measures that place services on a more sustainable footing. International experience shows how these can be at odds, especially if actions taken in the short term exhaust an already depleted workforce, or resolve Covid-19-specific problems but leave services less prepared for tomorrow's challenges.

